# Neuropathology of frontotemporal lobar degeneration: a review

**DOI:** 10.1590/S1980-57642013DN70100004

**Published:** 2013

**Authors:** Valéria Santoro Bahia, Leonel Tadao Takada, Vincent Deramecourt

**Affiliations:** 1 MD, PhD. Behavioral and Cognitive Neurology Unit, Department of Neurology, Hospital das Clínicas, University of São Paulo School of Medicine, São Paulo SP, Brazil.; 2 MD, Behavioral and Cognitive Neurology Unit, Department of Neurology, Hospital das Clínicas, University of São Paulo School of Medicine, São Paulo SP, Brazil.; 3 MD, PhD, Univ Lille Nord de France, Laboratory of Excellence DISTALZ, Memory Clinic, Histology and Pathology Department, Lille, France.

**Keywords:** frontotemporal lobar degeneration, pathology, TAU, TDP, FUS

## Abstract

Frontotemporal lobar degeneration (FTLD) is the second most common cause of presenile dementia. Three main clinical variants are widely recognized within the FTLD spectrum: the behavioural variant of frontotemporal dementia (bvFTD), semantic dementia (SD) and progressive non-fluent aphasia (PNFA). FTLD represents a highly heterogeneous group of neurodegenerative disorders which are best classified according to the main protein component of pathological neuronal and glial inclusions. The most common pathological class of FTLD is associated with the TDP-43 protein (FTLD-TDP), while FTLD-Tau is considered slightly less common while the FTLD-FUS (Fused in sarcoma protein) pathology is rare. In this review, these three major pathological types of FTLD are discussed.

## Abstract (pt)

A degeneração lobar frontotemporal (DLFT) é a segunda principal causa de demência pré-senil. Sob o diagnóstido de DLFT, há três principais diagnósticos clínicos: demência frontotemporal variante comportamental (DFTvc), demência semântica (DS) e a afasia progressiva não Fluente (APNF). A DLFT representa um grupo heterogêneo de desordens degenerativas que são classificadas de acordo com o componente proteico patológico das inclusões neuronais e gliais. A classe patológica mais comum das DLFT é associada com a proteína TDP-43 (DLFT-TDP), seguida pela DLFT-Tau, enquanto a DLFT-FUS é rara. Nesta revisão, nós iremos discutir os três principais subtipos patológicos da DLFT.

## INTRODUCTION

Frontotemporal lobar degeneration (FTLD) is the second most prevalent form of early-onset neurodegenerative dementia, after Alzheimer's disease (AD).^1-4^ In the United States, the prevalence of FTLD and primary progressive aphasia (PPA) among individuals between the ages of 45 and 64 years is estimated at around 15 to 22 per 100,000 person-years.^5^

FTLD includes a cluster of behavioral, cognitive and language disorders associated with degeneration of the frontal and anterior temporal lobes. The Consensus Criteria for FTLD^6^ distinguish three main clinical variants of FTLD: the behavioral variant of frontotemporal dementia (bvFTD), which is the most common clinical presentation, progressive non-fluent aphasia (PNFA), and semantic dementia (SD). There is considerable clinical overlap of these main variants of FTLD with Motor Neuron Disease (MND),^7^ corticobasal syndrome and other atypical extrapyramidal syndromes.

FTLD refers to a neuropathological entity characterized by degeneration in the frontal and/or anterior temporal cortices.

The new diagnostic consensus^8^ criteria for bvFTD require that for the diagnosis of possible bvFTD, three of the following behavioral/cognitive symptoms must be persistent or recurrent within the three first years of disease: behavioral disinhibition; apathy or inertia; loss of sympathy or empathy; perseverative, stereotyped or compulsive/ritualistic behavior; hyperorality and dietary changes; and, neuropsychological findings that include executive/generation deficits with relative sparing of memory and visuospatial functions. For the diagnosis of probable bvFTD, besides meeting criteria for possible bvFTD, the following must also be present: significant functional decline and neuroimaging findings consistent with bvFTD (i.e. frontal and/or anterior atrophy or hypometabolism). Finally, for bvFTD with definitive FTLD pathology, a diagnosis of possible or probable bvFTD is required, along with histopathological evidence of FTLD (on biopsy or at postmortem), or presence of a known pathogenic mutation.

The syndrome of Primary Progressive Aphasia (PPA) includes SD (also called semantic variant of PPA) and PNFA (non-fluent variant of PPA). PPA is defined as prominent language deficits during the initial stages of disease with insidious onset of symptoms.^9-11^

The diagnostic criteria for SD include: impaired confrontation naming, impaired single-word comprehension and at least three of the following: impaired object knowledge, surface dyslexia or dysgraphia, spared repetition and/or spared speech production (grammar and motor). Imaging must show involvement of the anterior temporal lobe. For the clinical diagnosis of PNFA, agrammatism in language production and/or apraxia of speech, and at least 2 of the three following features are required: impaired comprehension of syntactically complex sentences, spared single word comprehension and spared object knowledge. Neuroimaging typically shows predominant involvement of the left posterior frontoinsular region.^9-11^

FTLD is a heterogeneous disorder in relation to pathological and genetic findings.^12,13^ Neuropathologically, FTLD is classified into several subtypes, according to the main protein component of neuronal and glial abnormal inclusions and their distribution.^14^.^15^ Currently, three main proteins are associated with FTLD: the microtubule-associated protein Tau in FTLD-Tau, the transactive response DNA-binding protein 43 kD (TDP- 43) in FTLD-TDP, and the fused in sarcoma protein (FUS) in FTLD-FUS.^16-19^ FTLD-TDP is the most common subtype, representing about 50% of cases; FTLD-Tau is slightly less common (~45%) whereas the rarer FTLDFUS pathology is found in around 5% of cases.^20-23^

In clinicopathological studies, each FTD clinical phenotype has been associated with FTLD-tau, FTLDTDP and FTLD-FUS in different proportions. Based on pooled data from large neuropathological studies,^15^ bvFTD is associated with FTLD-TDP in around 50% of cases, with FTLD-tau in 40% of cases, and the remainder mostly with FTLD-FUS. When bvFTD presents with MND (FTD-MND), TDP-43 pathology is found in virtually all such cases. PNFA is linked to tau pathology in around 70% of cases. Typical SD, on the other hand, is due to TDP-43 pathology in more than 80% of cases.

A key concept in understanding the clinical presentation of bvFTD and PPA is that the anatomical pattern of degeneration – rather than the type of pathology – determines the symptoms.^24^ In bvFTD, early selective neuronal degeneration of Von Economo neurons and fork cells has been described.^25^ This selective neuronal loss has been observed in FTLD-tau, FTLD-TDP and FTLD-FUS, suggesting it occurs irrespectively of the abnormal protein. These neurons are found in the anterior insula, which together with the anterior cingulate cortex are early regions of degeneration in bvFTD.

In this review, we will cover the main pathological types of FTLD (namely, FTLD-tau, FTLD-TDP and FTLDFUS), describe the key finding in each type (and subtype), as well as discuss clinicopathological correlations.

## FTLD-TAU

The intraneuronal accumulation of filamentous, hyperphosphorylated microtubule-associated protein Tau is found in around 45% of cases of FTLD.^23^ Tau is a microtubule-associated protein that plays an important role in the assembly and stabilization of microtubules, and regulates axonal transport. Abnormal tau is hyperphosphorylated, assembled into insoluble filaments and then accumulates in neurons and/or glial cells.^27^ Tau is encoded by the *MAPT* gene, which is located at chromosome 17q21-22.^27^ *MAPT* mRNA can be alternatively spliced to include either three or four repeated amino-acid sequences that serve as microtubule- binding sites. One of the four repeated sequences is encoded by exon 10; inclusion of this sequence leads to 4 repeat (4R) tau isoforms, while exclusion leads to 3 repeat (3R) isoforms. Similar amounts of 3R and 4R tau are present in normal human brain, whereas pathological tau may be predominantly composed of 4R, 3R or both isoforms.^20,27^ Depending on the predominant tau isoform found in filaments, FTLD-tau subtypes are classified as 3R, 4R or 3R/4R tauopathies (Table 1).

**Table 1:** FTLD-tau and tau isoforms.

Predominant tau isoform	3R and 4R	3R	4R
Diseases	Neurofibrillary tangle dementia	Pick's disease	Corticobasal disease Progressive supranuclear paralysis Argyrophilic grain disease Multiple system tauopathy with dementia

While most FTLD-tau cases are sporadic, *MAPT* mutations are found in about 5% of FTLD cases.^27^ The mutations cause either a primary effect at the protein level or affect mRNA splicing sites, resulting in decreased ability to bind microtubules, increased tendency to form filaments, and/or altered ratio of tau 3R and/or 4R isoforms with accumulation of hyperphosphorylated tau filaments within neurons and glial cells.^27,28^ Currently, there are more than 45 described pathogenic mutations in *MAPT*, found in 9-21% of familial cases of FTD.^30,31^ *MAPT* mutations are exceptionally found in sporadic cases of FTLD. Phenotypic heterogeneity is common even within kindreds with the same *MAPT* mutation^29,32^ and different mutations have been associated with different tau isoforms^33^ and neuropathological findings.^26,34^

The most common subtypes of FTLD-tau^15,35^ include Pick's disease (PiD), corticobasal degeneration (CBD), progressive supranuclear palsy (PSP), and argyrophilic grain disease (AGD). Other subtypes of FTLD-tau are rare, but include: multiple system tauopathy with dementia (MSTD), neurofibrillary tangle predominant dementia (NFT-dementia) and white matter tauopathy with globular glial inclusions (WMT-GGI). Unclassifiable FTLD-tau is also acknowledged.

Among patients with bvFTD due to FTLD-tau, around 70% have Pick's disease, 20% have corticobasal degeneration (CBD) and most of the remainder have progressive supranuclear palsy (PSP) at neuropathological examination.^15,36^ As previously mentioned, FTLDtau is the most common neuropathological finding in PNFA.^37^ Among PNFA cases due to FTLD-tau, around 40% are diagnosed with PiD, 30% with PSP and 30% with CBD. SD is due to a tauopathy in only 20% of cases, most of which are classified as PiD or AGD.^15,37^

## PICK'S DISEASE

Intraneuronal argyrophilic inclusions (Pick bodies) and less specifically, ballooned cells (Pick cells) localized in the cytoplasm of neurons were first observed by Alois Alzheimer in the brains of patients with behavioral, language and apraxia symptoms and frontotemporal atrophy, previously described by Arnold Pick in 1889. It was only in the 1980s that tau protein was identified in these inclusions. For a long time, "Pick's disease" was used as a synonym of FTLD, but the term is currently only used to designate a neuropathological diagnosis of a tauopathy with Pick bodies and cells. PiD represents 5-8% of all FTLD cases.^21,26^ Pick bodies contain exclusively 3R tau and are particularly found in granule cells from the dentate gyrus, and pyramidal neurons in the hippocampus, temporal and frontal cortices.^34^

In a recent study, Piguet et al. (2011)^38^ identified 30 cases (but only 21 with sufficient clinical information) with pathological diagnosis of PiD from among 250 pathologically-confirmed FTLD cases collected over 16 years by two large brain banks. Of these cases, 13 had been diagnosed with bvFTD and 8 with language variant FTD (3 SD, 4 PNFA and 1 classified as "global").

## CORTICOBASAL DEGENERATION

CBD was initially described as an atypical parkinsonian disorder with signs of parietal cortical dysfunction, such as apraxia, cortical sensory deficits and alien limb. When neuropathological studies were done on CBD, it became clear that the phenotype associated with CBD was more heterogeneous than initially thought, and that other phenotypes also occurred. The "classic" phenotype is therefore now called corticobasal syndrome (CBS) and CBD is used to identify the neuropathological diagnosis. CBD has also been described in patients clinically diagnosed with PSP, bvFTD, PPA (particularly PNFA) and rarely, posterior cortical atrophy (PCA).^39^

The most specific neuropathological finding in CBD is astrocytic plaques.^21^ Swollen achromatic neurons may also be found, but those are not specific to CBD, being observable in other conditions.^21^ Other characteristic findings include: tau-positive threads, which are observed in the neocortex, subcortical white matter and basal ganglia, and oligodendroglial inclusions called coiled bodies.^34^ These inclusions are exclusively composed of 4R Tau.^40^

## PROGRESSIVE SUPRANUCLEAR PALSY

Clinically, PSP may present as a PSP syndrome (PSPS, also known as Richardson syndrome), bvFTD, PNFA, corticobasal syndrome (CBS) or pure akinesia with gait failure.^21^ Because there is significant clinical and neuropathological overlap between CBD and PSP, both 4R tauopathies are considered by some authors to lie within a disease spectrum.^21^

In PSP, neuronal loss and gliosis are most significant in the substantia nigra, the pallidum, anterior thalamus and subthalamic nucleus and when cortical pathology is present, lesions are usually found in primary motor and premotor cortices.^21^ Tufted astrocytes and globose neurofibrillary tangles are the most typical findings.^34^ Threads and coiled bodies are also found in PSP,^34^ but white matter pathology is less widespread than in CBD.^15^

## ARGYROPHILIC GRAIN DISEASE

AGD is the diagnosis in less than 10% of FTLD-tau,^15^ and to date, there is no single phenotype strongly associated with AGD. It may present as a late-onset amnestic syndrome (mean age at onset is 80 years), but a presentation similar to bvFTD has also been reported.^41^ Argyrophilic grains are also often found in non-demented elderly subjects, such as centenarians.^42^

Argyrophilic grains are oval, spindle or commashaped 4R tau-positive structures, and together with coiled bodies are the hallmark neuropathological findings in AGD.^43^ Macroscopically, AGD is usually associated with symmetrical temporal predominant atrophy, including the hippocampus. The neuropathological changes are typically confined to medial temporal lobe and limbic structures.^34^

In most cases (if not all) argyrophilic grains are found co-occurring with neuropathological changes of other neurodegenerative dementias, particularly the neurofibrillary tangles seen in Alzheimer's disease.^43^ Argyrophilic grains have also been observed in PiD, PSP, CBD, Parkinson's disease, dementia with Lewy bodies, among others.^43^ There has been some debate in the literature over the association between AGD and dementia, but some evidence suggests the presence of argyrophilic grains may lower the threshold for dementia in older individuals.^44^

## OTHER SUBTYPES OF FTLD-TAU

White matter tauopathy with globular glial inclusions and sporadic multiple system tauopathy with dementia are rare forms of FTLD-tau characterized by 4R tau-positive globular glial (astrocytic and oligodendroglial) inclusions that some argue are the same pathological entity.^45^ bvFTD, AD, PSP, CBS and MND have been reported as clinical phenotypes of patients diagnosed with this condition. Neurofibrillary tangle predominant dementia is characterized when neurofibrillary tangles (similar to those observed in Alzheimer's disease pathology) are found and neuritic plaques or amyloid deposits are absent.

## FTLD-TDP

In the 1990s, many of the cases without tau pathology were classified as "Dementia lacking distinctive histology" (DLDH),^5^ until new techniques of immunohistochemistry showed that many of these cases had ubiquitinated inclusions (denominated FTLD-U at the time).^47^ Researchers then tried to ascertain which pathogenic proteins were ubiquitinated, as the ubiquitin-proteasome system (UPS) is responsible for selective and timely protein turnover and is essential for proper cellular functions.^48^

In 2006, Neumann et al.^18^ demonstrated that the TAR-DNA binding protein 43 (TDP-43), a 43kDa protein, is the most common protein linked to ubiquitin in cases previously classified as FTLD-U, as well as the majority of sporadic (and some familial) amyotrophic lateral sclerosis (ALS). In accordance with this evidence, these cases came to be called FTLD-TDP. In 2006, two groups^49,50^ independently described mutations in the progranulin (GRN) gene as causative of autosomal dominant FTLD-TDP. The gene is located at 1.7Mb of *MAPT*, explaining the disease in tau negative familial cases yet with genetic linkage to the chromosomal region 17q21.

TDP-43 is a DNA, RNA, and protein binding implicated in the regulation of numerous processes, including transcription, splicing cell cycle regulation, apoptosis, microRNA biogenesis, mRNA transport to and local translation at the synapse and scaffolding for nuclear bodies.^51,52^ In pathological conditions, TDP-43 is displaced from the nucleus to the cytoplasm, hyperphosphorylated, ubiquitinated and cleaved to produce C-terminal fragments.^22,53^

TDP-43 proteinopathy is not unique to FTLD but is also a pathological hallmark of other neurodegenerative disorders, such as motor neuron disease (MND) with or without dementia, and Perry syndrome.^18^ TDP-43 positive inclusions are occasionally found in Alzheimer's disease, Parkinson dementia complex of Guam, and Lewy body disease. Consequently, some authors question whether abnormal aggregation of this protein is a cause or consequence of the neurodegenerative process.^54,55^ Up until 2011, there were two pathological classifications for FTLD-TDP.^56,57^ The Sampathu et al.^57^ classification was based on differential labeling of the pathology by a panel of monoclonal antibodies while the classification proposed by Mackenzie et al.^56^ prioritized the finding of the relatively specific clinicopathological correlations. However, in 2011 these authors et al. created a consensus classification system for FTLD pathology.^58^ In this harmonized criteria, the subtypes are denominated by letters in decreasing order of frequency, considering clinical and genetic correlations (Table 2).

**Table 2:** Harmonized classification for FTLD-TDP pathology (Adapted from Mackenzie et al., 2011)74.

Subtypes	Common phenotype	Pathological findings
A	bvFTD and PNFA	Numerous short DN and crescentic or oval NCI concentrated in neocortical layer 2. Moderate number of lentiform NII are common but inconsistent in this subtype.
B	bvFTD and MND with FTD	Moderate numbers of NCI, throughout all cortical layers with few DN.
C	SD and bvFTD	Predominance of elongated DN in upper cortical layers, with few NCI.
D	Familial IBMPFD	Numerous short DN and frequent lentiform NII.

bvFTD: behavioral variant frontotemporal dementia; PNFA: Progressive non-fluent aphasia; GRN: progranulin gene; MND: motor neuron disease; SD: semantic dementia; IBMPFD: inclusion body myopathy with Paget's disease of bone and frontotemporal dementia; VCP: valosin-containing protein gene.

**Consensus classifications.** FTLD-TDP type A is characterized by numerous short dystrophic neurites (DN), crescentic or oval neuronal cytoplasmatic inclusions (NCI), and lentiform neuronal intranuclear inclusions (NII) concentrated primarily in neocortical layer 2. Type B shows moderate numbers of NCI throughout all cortical layers, but very few DN. Type C has a predominance of elongated DN in upper cortical layers, with very few NCI. Type D refers to the pathology associated with Inclusion Body Myopathy with Paget's Disease of Bone and Frontotemporal Dementia (IBMPFD) caused by valosin-containing protein (VCP) mutations and is characterized by numerous short DN and frequent lentiform NII.^58^ Recently, some authors have suggested a new classification based on the molecular properties of TDP-43.^59^

In ALS, a progressive degenerative process affecting upper (cortical and brainstem structures) and lower motor neurons (spinal and bulbar), along with TDP-43 inclusions, can be found in these regions.^60^

*TARDBP* is the gene that encodes TDP-43 and is located on chromosome 1p^36^. There are 33 known pathogenic mutations (www.molgen.ua.ac.be/FTDmutations). These mutations are present in cases of sporadic or familial FTLD (with or without MND) and in cases of Amyotrophic Lateral Sclerosis (ALS).^61,62^ Borroni et al., 2010,^62^ evaluated the role of *TARDBP* mutations in 252 FTLD consecutive patients (153 bvFTD, 15SD, 22PNFA, and 62 CBS) and found only five mutations in patients with bvFTD and FTD-MND.

The main gene associated with autosomal dominant but also sporadic cases of FLTD-TDP (type A or B) is *C9ORF72*.^63^ These cases also exhibit cerebellar P62 positive inclusions in granular and molecular layers. The PGRN mutations typically lead to FTLD-TDP type A pathology.

## FTLD-FUS

In 2009, soon after the discovery that mutations in the FUS gene were causative of familial ALS^19,64^ and considering the significant genetic and pathologic overlap between FTLD and ALS, the Fused in Sarcoma (FUS) protein was identified as the third major abnormal protein in FTLD.^65-67^ FTLD-FUS is found in about 5% of FTLD cases.^67-69^

Similarly to TDP-43, FUS is a DNA and RNA-binding protein involved in gene expression, transcription regulation, RNA splicing, transport and translation.^69,70^ Both shuttle between the nucleus and cytoplasm, although are mainly expressed in the nucleus under normal conditions. Mutations in *FUS* have been reported in approximately 4% of familial ALS and <1% of sporadic cases, but are exceptional in bvFTD.

FUS immunoreactive inclusions may be found in neurons and glial cells, and the morphology and distribution of inclusions define the three subtypes of FTLDFUS: atypical FTLD-U (aFTLD-U), basophilic inclusions body disease (BIBD), and neuronal intermediate filament inclusion disease (NIFID) are now recognized as subtypes of FTLD-FUS.^35^

Atypical FTLD-U is associated with distinct clinical, radiological and neuropathological features.^68,72-74^ The clinical presentation is of early onset sporadic bvFTD (mean age at onset around 40 years and range 28-66 years). Psychotic symptoms are frequent, and family history is typically negative (*FUS* mutations have not yet been reported in FTLD-U). A peculiar neuroimaging finding is caudate atrophy, although frontotemporal atrophy is also observed. Neuropathologically, aFTLD-U is characterized by FUS-immunoreactive neuronal intranuclear and cytoplasmic inclusions.^67^ Inclusions are most commonly found in the frontal and temporal neocortex, and hippocampus. Intranuclear neuronal inclusions have a unique appearance of elongated straight, curved, vermiform or ring-like structures. Oligodendroglial inclusions are also present, although subcortical pathology is less prominent than in the other FTLDFUS subtypes.^74^

Unlike aFTLD-U, the clinical presentations of BIBD and NIFID are heterogeneous. NIFID is most commonly associated with bvFTD, but PNFA, FTD-MND and primary lateral sclerosis with CBS (PLS/CBS) have also been reported.^15,70,74^ NIFID is characterized by neuronal inclusions that are immunoreactive for intermediate filaments, as well as FUS-positive NCIs, NIIs, and GCIs. BIBD is rare and has been reported in cases diagnosed clinically as bvFTD, FTD-MND, PSP and PLS/CBS (as well as pure MND).^15^ The nomenclature derives from the basophilic cytoplasmic inclusions observed on haematoxylin and eosin staining, which are strongly positive for FUS immunohistochemistry.^65^ NIIs are rare in BIBD, but GCIs are common.

**Figure 1:**
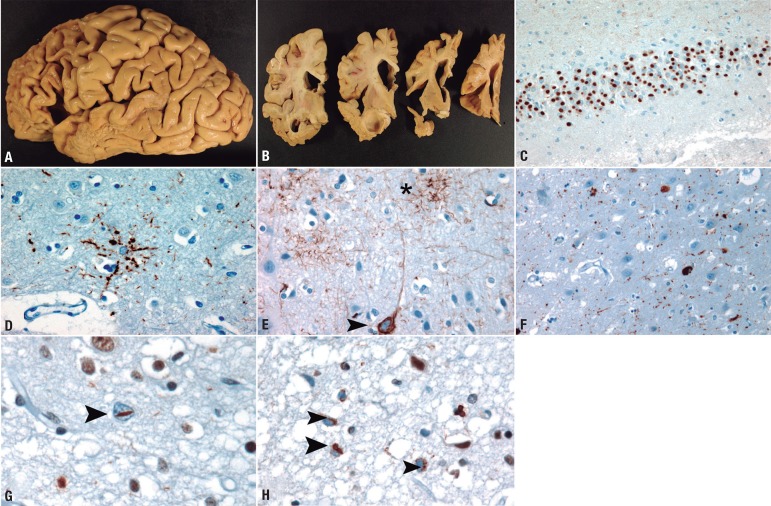
[A and B] Typical frontotemporal atrophy in a case of Pick's disease. [C] Pick bodies in dentate neurons of the hippocampus (Tau immunochemistry). [D] Astrocytic plaque in a CBD case (frontal cortex, Tau immunochemistry). [E] Tufted astrocyte (asterisk) and neurofibrillary tangle in a case of PSP (primary motor cortex,Tau immunochemistry). [F] Grains and neurofibrillary tangles in a case of AGD (hippocampus, Tau immunochemistry). [G] Lentiform neuronal intranuclear inclusion in a case of FTLD-TDP type A (asterisk, frontal cortex, TDP-43 immunochemistry). [H] Neuronal cytoplasmic inclusions in a case of FTLD-TDP type B (arrowheads, primary motor cortex, TDP-43 immunohistochemistry).

## FTLD-UPS

The major abnormal protein remains unknown in a few cases of FTLD. Some of these cases test positive for ubiquitin and p62 staining but negative for tau, TDP-43 and FUS, and are thus called FTLD-UPS (due to involvement of the ubiquitin-proteasome system).^22,35,74^ Most FTLD-UPS cases are associated with CHMP2B mutations, a form of genetic FTD identified in Denmark and Belgium. FTLD without inclusions (FTLD-ni) is now considered rare.^15^

In conclusion, the pathology of FTLD is heterogeneous and remains highly unpredictable during the patient's life. Therefore, the diagnosis of definite FTLD can only be obtained after post mortem examination of the brain or with the identification of a pathogenic mutation associated with FTLD pathology. In familial FTLD, autopsy findings are very useful to identify the causative gene. Large post mortem studies are still needed to increase knowledge on the clinical-pathological correlations of FTLD.
